# Dynamic Fluorescence Microscopy of Cellular Uptake of Intercalating Model Drugs by Ultrasound-Activated Microbubbles

**DOI:** 10.1007/s11307-016-1042-x

**Published:** 2017-02-17

**Authors:** B.H.A. Lammertink, R. Deckers, M. Derieppe, I. De Cock, I. Lentacker, G. Storm, C.T.W. Moonen, C. Bos

**Affiliations:** 1 Imaging Division, UMC Utrecht, Heidelberglaan 100, 3584 CX Utrecht, The Netherlands; 2 Department of Pharmaceutics, Ghent University, Ghent, Belgium; 3 Pharmaceutics Department, Utrecht University, Utrecht, The Netherlands; 4 Targeted Therapeutics, MIRA Institute, University of Twente, Enschede, The Netherlands

**Keywords:** Model drug, Fluorescence, Confocal microscopy, Ultrasound, Microbubbles, Drug delivery

## Abstract

**Purpose:**

The combination of ultrasound and microbubbles can facilitate cellular uptake of (model) drugs via transient permeabilization of the cell membrane. By using fluorescent molecules, this process can be studied conveniently with confocal fluorescence microscopy. This study aimed to investigate the relation between cellular uptake and fluorescence intensity increase of intercalating model drugs.

**Procedures:**

SYTOX Green, an intercalating fluorescent dye that displays >500-fold fluorescence enhancement upon binding to nucleic acids, was used as a model drug for ultrasound-induced cellular uptake. SYTOX Green uptake was monitored in high spatiotemporal resolution to qualitatively assess the relation between uptake and fluorescence intensity in individual cells. In addition, the kinetics of fluorescence enhancement were studied as a function of experimental parameters, in particular, laser duty cycle (DC), SYTOX Green concentration and cell line.

**Results:**

Ultrasound-induced intracellular SYTOX Green uptake resulted in local fluorescence enhancement, spreading throughout the cell and ultimately accumulating in the nucleus during the 9-min acquisition. The temporal evolution of SYTOX Green fluorescence was substantially influenced by laser duty cycle: continuous laser (100 % DC) induced a 6.4-fold higher photobleaching compared to pulsed laser (3.3 % DC), thus overestimating the fluorescence kinetics. A positive correlation of fluorescence kinetics and SYTOX Green concentration was found, increasing from 0.6 × 10^−3^ to 2.2 × 10^−3^ s^−1^ for 1 and 20 μM, respectively. Finally, C6 cells displayed a 2.4-fold higher fluorescence rate constant than FaDu cells.

**Conclusions:**

These data show that the temporal behavior of intracellular SYTOX Green fluorescence enhancement depends substantially on nuclear accumulation and not just on cellular uptake. In addition, it is strongly influenced by the experimental conditions, such as the laser duty cycle, SYTOX Green concentration, and cell line.

**Electronic supplementary material:**

The online version of this article (doi:10.1007/s11307-016-1042-x) contains supplementary material, which is available to authorized users.

## Introduction

The combination of ultrasound (US) and microbubbles (MB) is a non-invasive technique to enhance local drug concentration *in vivo* [1], without encapsulating the drug [2] or altering its physicochemical properties [3]. Ultrasound and microbubble (USMB)-induced cavitation has been demonstrated to transiently disturb the integrity of plasma membranes, increasing its permeability and thereby enhancing intracellular (model) drug uptake [4–6].

Several mechanisms have been proposed to explain this phenomenon, including pore formation [7, 8] and upregulation of endocytosis [9, 10]. It has been demonstrated that the size of the (model) drug [9] and the acoustic pressure used in the experiment [10] affect the mode of cellular uptake.

Uptake of fluorescent model drugs can be assessed on a single-cell level using confocal fluorescence microscopy, allowing real-time monitoring of the intracellular spatial distribution of the model drug, in contrast to analytical techniques such as high-performance liquid chromatography. By analyzing single cells, confocal fluorescence microscopy can detect the heterogeneity of the underlying microbubble-cell interactions, as opposed to the cell population-based analytical techniques.

Intercalating fluorescent model drugs, such as SYTOX Green or propidium iodide (PI), are extensively used to study USMB-induced intracellular drug delivery [11, 12]. They are hydrophilic molecules that are normally not taken up by cells and become strongly fluorescent upon binding nucleic acids after entering the intracellular domain [16]. These properties make these compounds convenient for studying USMB-induced membrane permeabilization and drug uptake using conventional research techniques, e.g., fluorescence microscopy and flow cytometry [13–15].

Dedicated setups are increasingly being used to dynamically monitor USMB-induced cellular internalization of these model drugs in real-time by confocal fluorescence microscopy, aiming to give a quantitative description of the uptake kinetics [16–19]. However, when monitoring the kinetics of cellular internalization, results should be interpreted with caution, as the fluorescence intensity of these intercalating model drugs not only depends on cellular uptake but also on nucleic acid binding. In other words, the fluorescence intensity is no longer strictly proportional to the intracellular concentration, which may result in misinterpretation of the observed data.

Therefore, the research in this manuscript aimed to increase our understanding of the kinetics of fluorescence enhancement following USMB-induced cellular internalization of intercalating model drugs. This allows to improve the experimental design of future experiments as well as to guide the interpretation of observed results.

To this end, the study characterized the fluorescence intensity enhancement upon USMB-induced intracellular SYTOX Green uptake *in vitro*, as a function of the experimental parameters. USMB-induced SYTOX Green uptake was monitored in high spatiotemporal resolution in single cells using confocal fluorescence microscopy. In addition, the fluorescence kinetics were studied in a population of cells to assess the effect of laser duty cycle, SYTOX Green concentration, and cell line.

## Materials and Methods

### Cell Culture

Human melanoma (BLM) cells [20] were cultured in Dulbecco’s modified Eagle’s medium (DMEM) with Nutrient Mixture F12 (Gibco, Merelbeke, Belgium), supplemented with 10 % fetal bovine serum (FBS) (Hyclone, Thermo Scientific, MA, USA), 20 U/ml penicillin-streptomycin (Gibco), 2 mM l-glutamine (Gibco), and 10 mM HEPES (Sigma-Aldrich®). Human pharynx squamous cell carcinoma (FaDu) cells (ATCC® HTB-43™, LGC Standards GmbH, Wesel, Germany) were cultured in high-glucose DMEM (Sigma-Aldrich®, St. Louis, MO, USA), supplemented with 10 % (*v/v*) FBS (Sigma-Aldrich®) and 1 % non-essential amino acids (Sigma-Aldrich®). Rat glioma (C6) cells (ATCC® CCL-107™) were maintained in low-glucose DMEM (Sigma-Aldrich®) supplemented with 10 % FBS. Cells were cultured in standard cell culture flasks in a humidified incubator at 5 % CO_2_ and 37 °C.

Ultrasound experiments with BLM cells were performed in OptiCells™ (Nunc, Thermo Scientific, MA, USA), wherein 1.3 × 10^6^ cells were plated 1 day prior to the experiment. For ultrasound experiments with FaDu or C6 cells, 1 × 10^6^ cells were seeded into CLINIcell® cell culture chambers (Mabio, Tourcoing, France) 2 days prior to the experiment, to ensure a confluent cell monolayer during the experiment. CLINIcells® were coated with Poly-l-Lysine (Sigma-Aldrich®) before cell seeding for proper cell attachment.

### Microbubbles

For the experiments with BLM cells, microbubbles composed of a DPPC and DSPE-PEG shell-encapsulating C_4_F_10_ gas were prepared as described previously [10]. They were freshly prepared on the experimental day and kept for a maximum of 4 h. In this setup, 40 μl of microbubbles was added to a 10-ml medium just before injection into the OptiCell™. In the US experiments with FaDu and C6 cells, the ultrasound contrast agent SonoVue™ (Bracco, Milan, Italy) was used, as described previously [12].

### Chemicals

SYTOX Green (Life Technologies™ Europe BV, Bleiswijk, Netherlands; excitation (Ex)/emission (Em) = 504/523 nm) was used as a model drug. SYTOX Green shows little native fluorescence, but upon binding to nucleic acids, the fluorescence intensity enhances >500-fold. PI, another commonly used fluorescent model drug, was used to compare the fluorescence kinetics between the two model drugs. PI (Thermo Fisher Scientific) fluorescence intensity enhances 30-fold upon binding to nucleic acids. CellMask™ Deep Red plasma membrane stain (Thermo Fisher Scientific, Waltham, MA, USA; Ex/Em = 649/666 nm) was used to visualize the plasma membrane in the swept field confocal microscopy experiments. In the same experiments, Hoechst 33342 (Sigma-Aldrich®; Ex/Em = 350/461 nm) was used to stain cell nuclei, which allowed for segmentation of the nucleus in the data analysis.

### Ultrasound Equipment

**Fig. 1.**
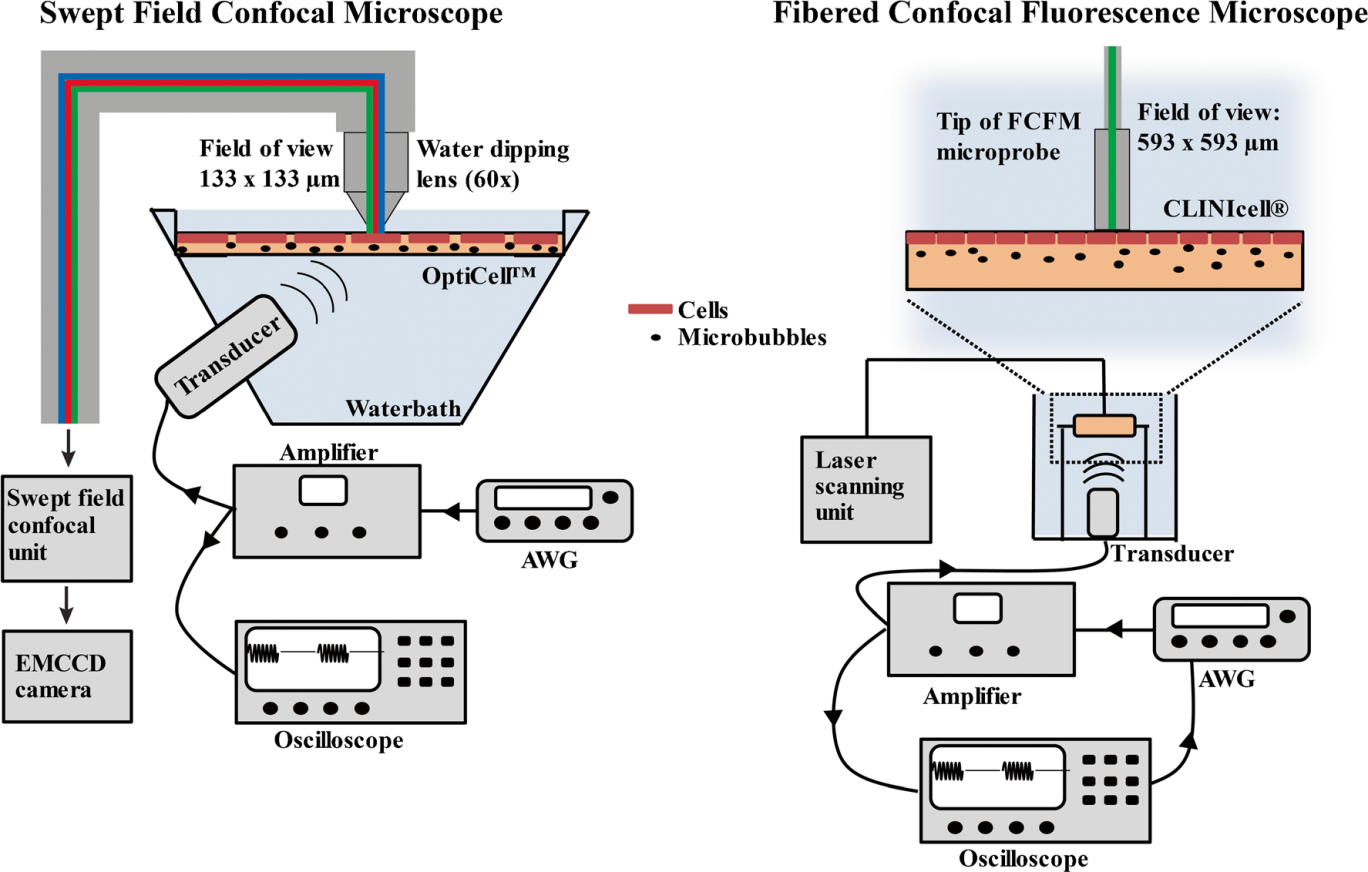
Schematic representation of the confocal microscopy systems and ultrasound setups used in this study.

Two ultrasound setups were used in these studies, each dedicated to its own confocal microscopy system. First, an ultrasound setup mounted on a swept field confocal microscope for real-time confocal recordings was used as described previously (Fig. 1, left panel) [10]. In this setup, BLM cells were sonicated for 5 s with 1.0 MHz ultrasound at 15 % duty cycle (DC), 1 kHz pulse repetition frequency (PRF), and 100 kPa peak negative pressure (PNP), as was calibrated with a needle hydrophone. These settings were based on previous work with this cell line [10].

A second ultrasound setup was used in combination with a fibered confocal fluorescence microscope, as reported before [16]. Here, C6 and FaDu cells were exposed to 1.5 MHz ultrasound for 5 s, at 10 % DC and 1 kHz PRF. These settings were based on previous experience with C6 cells [16]. For SYTOX Green uptake experiments, FaDu or C6 cells were exposed to 350, 600 and 850 kPa PNP, as was calibrated by a fiber optic hydrophone (Fibre-optic Hydrophone System, Precision Acoustics).

### High-Resolution Monitoring of Ultrasound-Induced SYTOX Green Uptake in Single Cells

Ultrasound-induced SYTOX Green uptake in BLM cells was monitored in detail using a swept field confocal microscope [10]. Cellular plasma membranes and nuclei were stained with 4 μg/ml CellMask™ Deep Red and 20 μM Hoechst, respectively, for 20 min prior to the uptake experiment. Then, 10 ml Opti-MEM® (Gibco) with 50 μl microbubbles and 2 μM SYTOX Green was added to the OptiCell™, which was placed in the water bath, such that the bubbles to ascended towards the cells (Fig. 1). Recordings started 10 s prior to US exposure, and images were acquired for 9 min. During post-processing of the data, the Hoechst signal was used to segment the nuclei, while a second region of interest was drawn manually in the cytosol. Subsequently, SYTOX Green fluorescence enhancement in the nucleus and the cytosol were analyzed in the Icy software [21]. Nuclear SYTOX Green signal was averaged every 20 s to minimize the effect of focal plane drift that occurred during acquisition.

### Monitoring of USMB-Induced SYTOX Green Uptake in a Population of Cells

SYTOX Green uptake following USMB treatment was monitored in a population of cells using a fibered confocal fluorescence microscope (FCFM) (Cellvizio®, Mauna Kea Technologies, Paris, France), as previously described [22]. The FCFM microprobe has a field of view of 593 × 593 μm, and a 488 nm laser was used to excite SYTOX Green at 0.5 mW laser power. As SYTOX Green fluorescence signal enhancement continued for 10–30 min, snapshots were recorded every 30 s for 30 min.

### Influence of Laser-Induced Photobleaching on Fluorescence Kinetics

First, the effect of photobleaching on the measured fluorescence intensity following USMB-induced SYTOX Green uptake was studied in FaDu cells. Cells were exposed to ultrasound in the presence of 2 μM SYTOX Green and SonoVue™ microbubbles. SYTOX Green uptake was recorded using the FCFM, while the laser output was switched off between acquisitions (3.3 % duty cycle, henceforth termed pulsed laser) or with a laser that was continuously on (100 % duty cycle).

In order to separate photobleaching and uptake, confluent FaDu cells were chemically permeabilized with 0.5 % Triton X-100 for 1 min and then washed and fixed in 4 % PFA during 15 min on ice. Subsequently, cells were incubated with 2 μM SYTOX Green for 30 min. Next, the staining solution was removed and fresh SYTOX Green-free PBS was added. The fluorescence signal of the SYTOX Green positive cells was recorded using the FCFM with pulsed and continuous laser.

### Concentration-Dependent Fluorescence Kinetics

To assess the influence of dye concentration on the fluorescence kinetics, FaDu cells were exposed to ultrasound in the presence of 1, 2, 5, or 10 μM SYTOX Green and SonoVue™ microbubbles. To compare the fluorescence kinetics of SYTOX Green with PI, similar experiments were performed with 30 μM PI, which was the minimum concentration required to obtain sufficient signal for kinetics quantification. Signal intensities of SYTOX Green or PI positive cells were recorded with pulsed laser on the FCFM. The relation between the fluorescence rate constant and SYTOX Green concentration was tested by linear regression.

### SYTOX Green Uptake in Chemically Permeabilized Cells

The fluorescence kinetics of SYTOX Green following internalization in chemically permeabilized cells was measured by spectrofluorometry. To reach a confluent monolayer during the experiment, 12 × 10^3^ C6 cells or 10 × 10^3^ FaDu cells were seeded per well of a 96-well plate and grown for 2 or 3 days, respectively. Then, cells were washed by PBS and permeabilized by adding 0.5 % Triton X-100 for 1 min. After washing, 1, 2, 5, 10, or 20 μM SYTOX Green was added and the well plate was immediately placed in the spectrofluorometer (FP8300, Jasco, Easton, MD, USA). Fluorescent signal enhancement (excitation 504 nm, emission 530 nm) was measured every 2 min in FaDu cells and every minute in C6 cells for a total of 180 min. The fluorescence signal of the samples was corrected for the negative control, i.e., cells with PBS only. The relation between the fluorescence rate constant and SYTOX Green concentration was tested by linear regression.

### Cell Viability after USMB Exposure

Viability following USMB exposure was measured via MTS assay in FaDu and C6 cells. In short, 1 × 10^6^ cells were seeded into coated CLINIcells® 2 days before the experiments. Then, a mixture of 7 % (*v*/v) SonoVue™ microbubbles in 9.5 ml Opti-MEM® was added to the cells, and the complete cell monolayer was exposed by sliding the CLINIcell® over the ultrasound beam using a guidance frame [14]. After allowing cells to recover for 4 h [12], they were harvested by trypsinization, counted, diluted, and seeded into a 96-well plate. After 3 days, medium was refreshed and 20 μl of MTS dye was added (CellTiter 96® AQueous One Solution Cell Proliferation Assay, Promega, Leiden, Netherlands). Cells were incubated at 37 °C, and absorbance was measured at 492 nm using a spectrophotometer (Biochrom EZ Read 400, Isogen Life Sciences, Utrecht, Netherlands). The optical density of the samples was corrected for the background signal, i.e., medium and MTS dye without cells.

### Data Analysis

To obtain fluorescence kinetics insensitive to motion, the individual cells in the FCFM data were segmented and tracked over the 30 min acquisition using MATLAB (MathWorks, USA), to determine the photobleaching rate constant *k*_pb_ and the fluorescence rate constant *k*_f_, similar to Derieppe *et al.* [22]. For a detailed description of this method, the reader is referred to the “Electronic Supplementary Material” of this manuscript.

Statistical analysis was performed in GraphPad Prism (La Jolla, CA, USA). Means were compared by ANOVA and Tukey’s multiple comparison test.

## Results

USMB-induced SYTOX Green uptake was monitored in high spatiotemporal resolution in BLM cells using a swept field confocal microscope. In addition, the fluorescence kinetics were studied in a population of cells as a function of laser duty cycle, dye concentration, and cell line using the fibered confocal fluorescence microscope.

### High-Resolution Imaging of USMB-Induced SYTOX Green Uptake in Single Cells

**Fig. 2.**
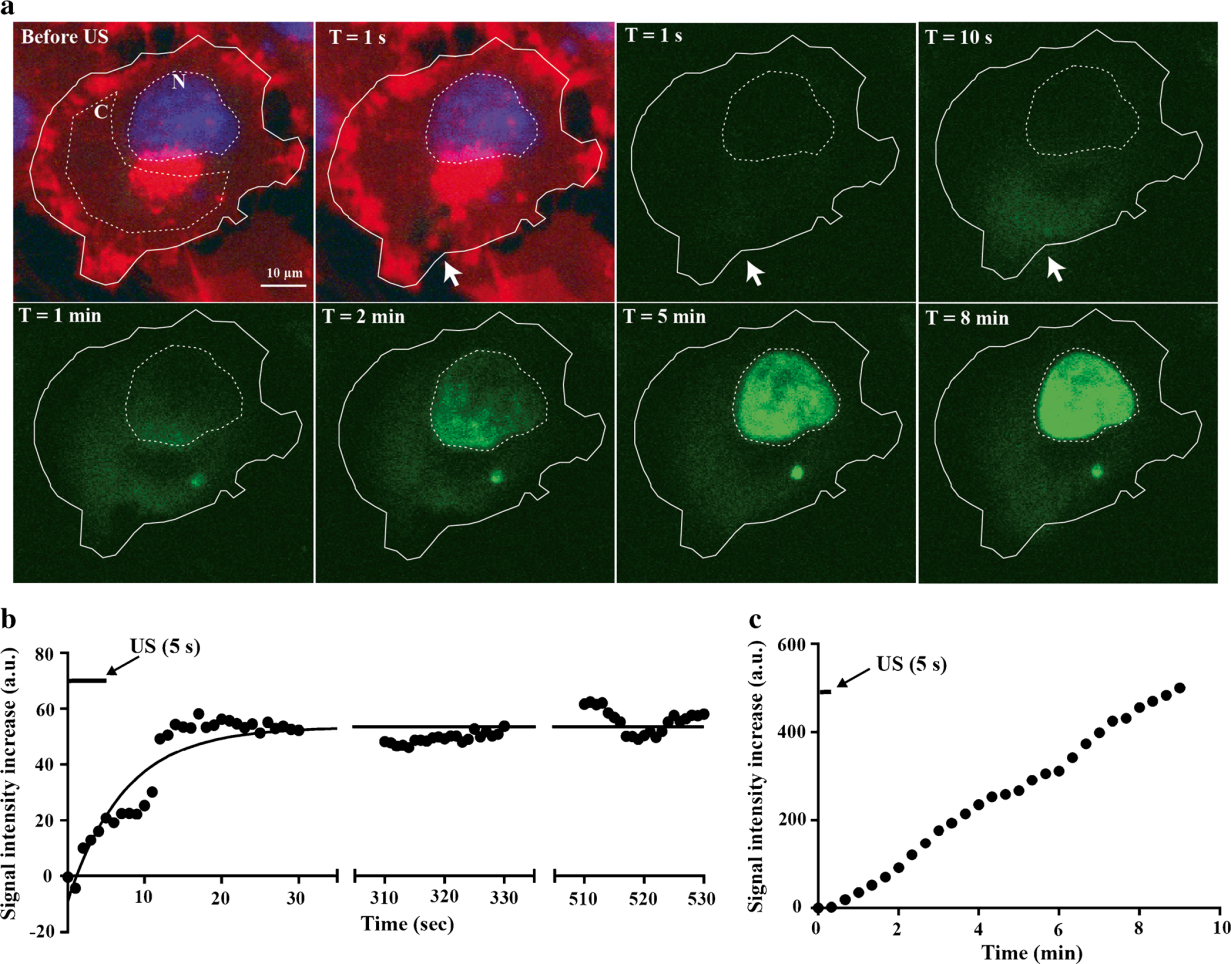
USMB-induced SYTOX Green uptake. **a** Time series of confocal images. Cell membrane and nucleus were labeled in *red* and *blue* to show the cell morphology before ultrasound was turned on and the pore created by ultrasound (*white arrow*). SYTOX Green uptake is monitored over the next 8 min (shown in *green*). Two regions of interest were drawn in the cell to analyze the SYTOX Green signal in these compartments: *C* marks the cytosolic region of interest, and *N* marks the nucleus. Ultrasound was turned on for 5 s at *T* = 0. **b** Signal intensity time curve of SYTOX Green in the cytosolic region of interest of the BLM cell shown in (**a**). **c** Nuclear signal intensity time curve of SYTOX Green in the same cell. For the colored version of the figures, the reader is referred to the online manuscript.

Swept field confocal microscopy allowed to qualitatively assess the relation between SYTOX Green uptake and fluorescence intensity. Upon ultrasound exposure, a membrane pore is created (Fig. 2a). The intracellular signal around the pore increases in the next 10 s, suggesting that the pore facilitates a local entry of SYTOX Green into the cell. During the subsequent 50 s, SYTOX Green fluorescence spreads from the pore area further into the cytosol, indicating diffusion through the cytosol. After about 1 min, the fluorescence signal also increases in the nucleus, starting at the side of the membrane pore. Over the next minutes, the fluorescence signal of the nucleus further enhanced and became more homogeneous, most likely representing an increase in SYTOX Green molecules binding to DNA after entering the nucleus. Image analysis demonstrated that mean fluorescence intensity of SYTOX Green in the cytosol increased over a time period of about 15 s after US was turned on, before it reached a maximum signal plateau (Fig. 2b), while the signal intensity in the nucleus kept increasing during the whole 9-min acquisition (Fig. 2c).

### Influence of Laser-Induced Photobleaching on Fluorescence Kinetics

**Fig. 3.**
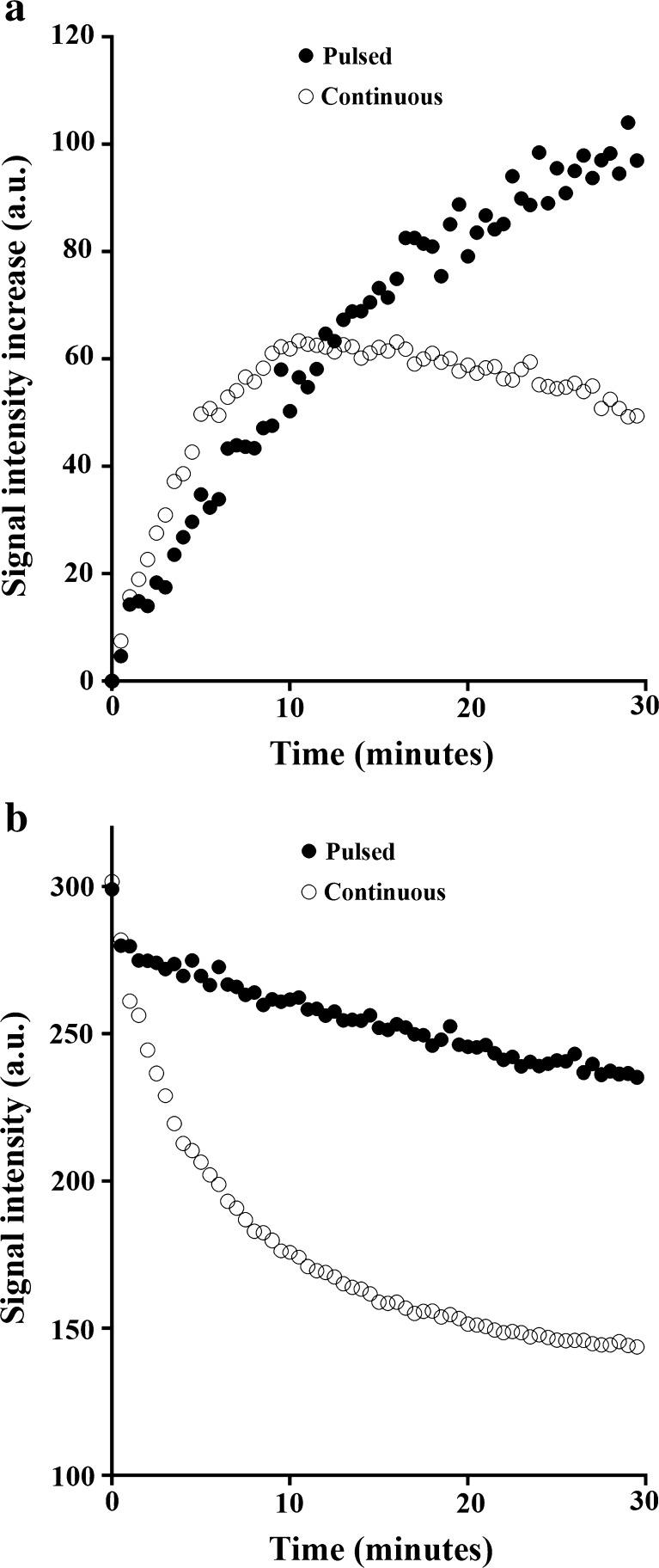
Fluorescence intensity of representative FaDu cells exposed to 2 μM SYTOX Green as a function of time exposed to a pulsed (laser duty cycle of 3.3 %) or continuous laser. **a** Fluorescence intensity of FaDu cells following ultrasound-induced SYTOX Green uptake. **b** Fluorescence intensity of permeabilized, fixed, and SYTOX Green-stained FaDu cells over time of acquisition.

The effect of photobleaching on the fluorescence kinetics was investigated by monitoring ultrasound-induced SYTOX Green uptake using the pulsed mode or continuous mode of the FCFM fluorescence laser. While other experimental conditions were identical, continuous laser led to a different signal intensity profile compared to pulsed laser (Fig. 3a) and fluorescence rate constants as fitted with the two-compartment model differed significantly, 1.02 (±1.09) × 10^−3^ s^−1^ for pulsed laser and 6.53 (±3.29) × 10^−3^ s^−1^ for continuous laser, respectively.

When trying to correct for photobleaching by using a three-compartment model to fit the fluorescence intensity, an average fluorescent rate constant of 0.34 (±0.21) × 10^−3^ s^−1^ is obtained, which is substantially different from the pulsed laser results. While the photobleaching correction by the three-compartment model somewhat improved the accuracy of the fluorescence kinetics, it did not completely correct for photobleaching in practice (Table 1).

**Table 1:** Photobleaching rate constants *k*_pb_ and fluorescence intensity rate constants *k*_f_ of SYTOX Green in FaDu cells

	Pulsed laser (10^−3^ s^−1^)	Continuous laser (10^−3^ s^−1^)	Ratio	Significance (*p*)
*k*_pb_	0.41 (±0.35)	2.63 (±0.31)	6.4	<0.0001
*k*_f_ 2CM	1.02 (±1.09)	6.53 (±3.29)	6.4	<0.0001
*k*_f_ 3CM	n/a	0.34 (±0.21)		

Values represent mean ± standard deviation

*2CM* two-compartment model, *3CM* three-compartment model, *n/a* not applicable

The laser duty cycle also affected the signal intensity profiles of chemically permeabilized, SYTOX Green positive cells (Fig. 3b). Although both cells had about the same fluorescence intensity at the start of the recording, the cellular SYTOX Green signal intensity decreased much faster when the cell was exposed to continuous laser, compared to pulsed laser, with a photobleaching rate constant *k*_pb_ that was 6.4-fold higher for the continuous laser (Table 1). This is equivalent to the 6.4-fold higher fluorescence rate constant *k*_f_ of the continuous laser compared to the pulsed laser, suggesting that the different fluorescence intensity time curves of Fig. 3a are primarily the result of photobleaching.

### Concentration-Dependent Fluorescence Kinetics

**Fig. 4.**
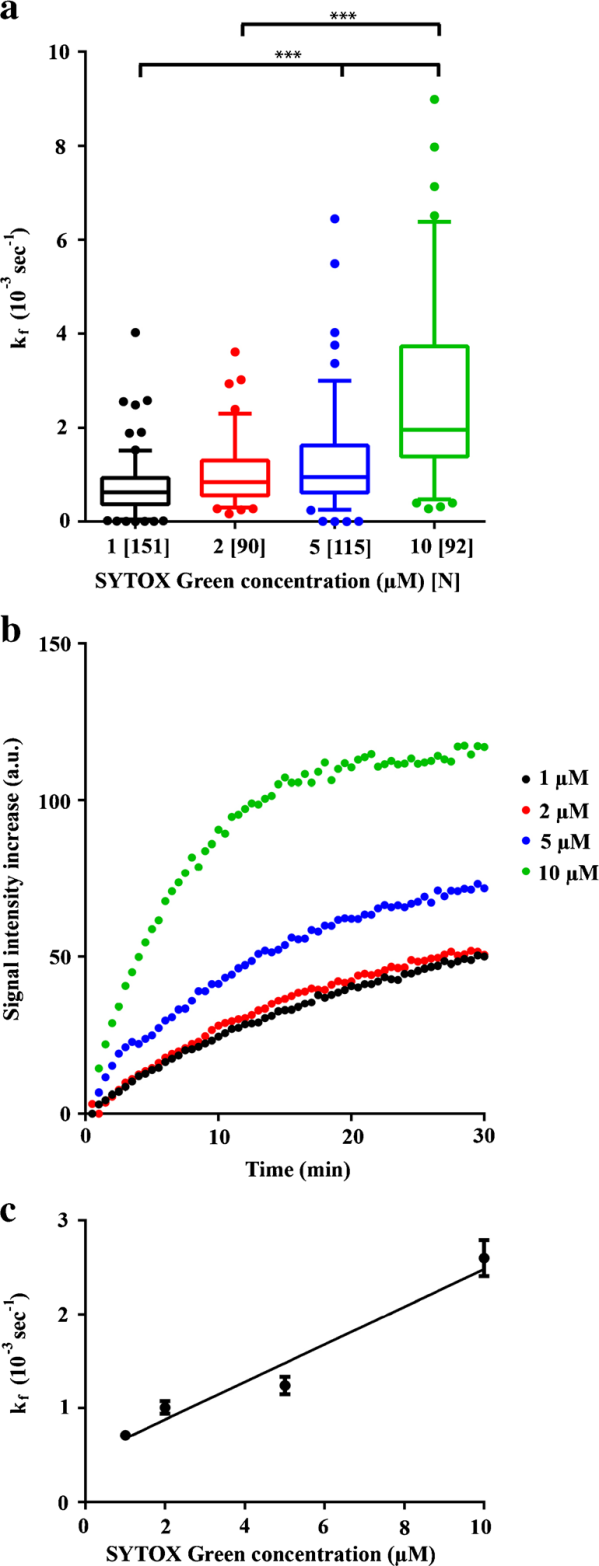
**a** Fluorescence rate constants (*k*_f_) as a function of SYTOX Green concentration. *Whiskers* represent the 5–95 percentile and *N* the number of cells per group. **b** Fluorescence signal intensity as a function of time for representative cells that were exposed to ultrasound in the presence of 1, 2, 5, or 10 μM SYTOX Green. **c** Scatterplot of *k*_f_ as a function of SYTOX Green concentration. *Symbols* represent mean ± SEM. The *three asterisks* indicate *p* < 0.001.

To investigate the influence of dye concentration on the fluorescence kinetics, FaDu cells were exposed to USMB in the presence of different concentrations of SYTOX Green, while monitoring uptake in the cell population with FCFM, using the pulsed laser to minimize photobleaching. Linear regression demonstrated that the fluorescence rate constant *k*_f_ significantly increased with SYTOX Green concentration (*p* < 0.05; Fig. 4a). The fluorescence kinetics showed a linear relation with the concentration, described by *k*_f_ = 0.2 (±0.03) × 10^−3^ s^−1^ μM^−1^. In addition, increasing SYTOX Green concentrations resulted in higher signal intensities (Fig. 4b), indicating that for the concentrations evaluated, the available sites for DNA-SYTOX Green binding, which mainly determines the fluorescence signal intensity observed in the nuclei, were not saturated (Fig. 4b).

### SYTOX Green Fluorescence Kinetics in Chemically Permeabilized FaDu Cells

**Fig. 5.**
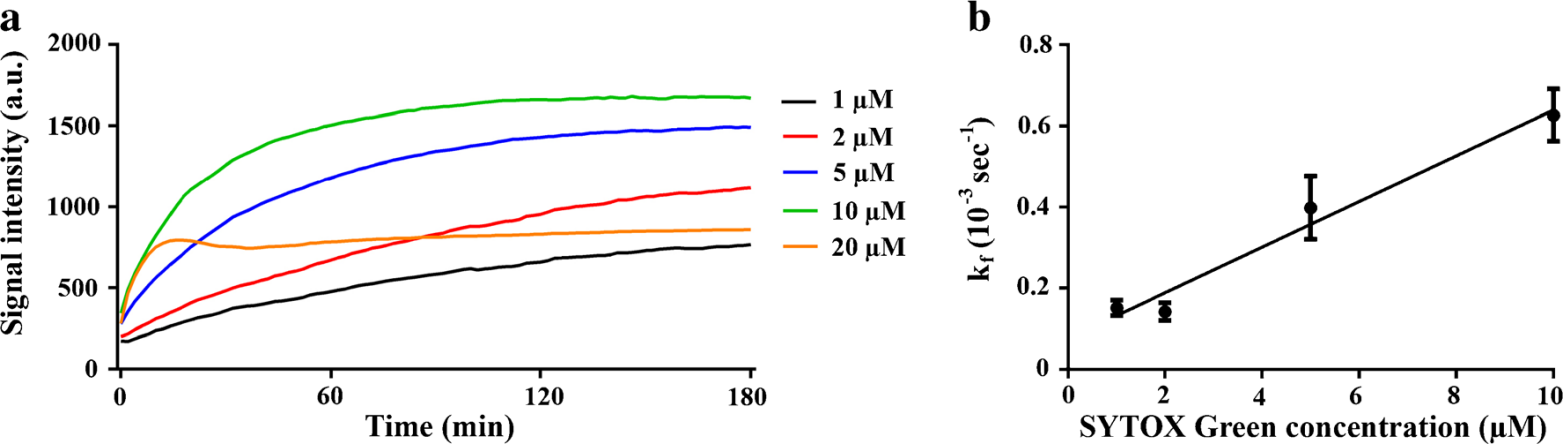
SYTOX Green uptake and fluorescence signal enhancement of chemically permeabilized FaDu cells. **a** Average SYTOX Green signal intensity over time after addition of 1, 2, 5, 10, or 20 μM to permeabilized cells (*N* = 4). **b** Fluorescence rate constants (*k*_f_) of signal intensity after addition of different SYTOX Green concentrations to permeabilized cells as a function of SYTOX Green concentration. *Symbols* represent mean ± SEM (*N* = 4).

To investigate the relation between SYTOX Green concentration and fluorescence kinetics in a model of membrane permeability where the effect of pore resealing after USMB-induced membrane pores is excluded, FaDu cells were chemically permeabilized and fluorescence intensity was measured over time in a cell population by fluorescence spectrometry. The fluorescence signal enhancement was faster with higher SYTOX Green concentrations (Fig. 5a). In FaDu cells exposed to 1 or 2 μM SYTOX Green, fluorescence intensity kept increasing for the duration of the measurement, i.e., 3 h. Similar to the ultrasound experiments, increasing dye concentration resulted in faster signal enhancement and enhanced maximal fluorescence intensity, except for the 20 μM SYTOX Green, where we suspect that quenching occurred. Up to 10 μM, the fluorescence rate constant *k*_f_ correlated linearly with SYTOX Green concentration (Fig. 5b), with a concentration dependence of *k*_f_ of 0.056 (±0.007) × 10^−3^ s^−1^ μM^−1^. These fluorescence rate constants are much lower compared to the fluorescence rate constants following USMB-induced permeabilization. This may suggest that the membrane integrity restored faster in USMB-exposed cells compared to chemically permeabilized cells. However, caution should be taken when comparing the fluorescence kinetics between USMB and chemical membrane permeabilization, because the method of permeabilization and the analytical technique differed between the experiments.

### Cell Line-Dependent Fluorescence Kinetics and Viability

**Fig. 6.**
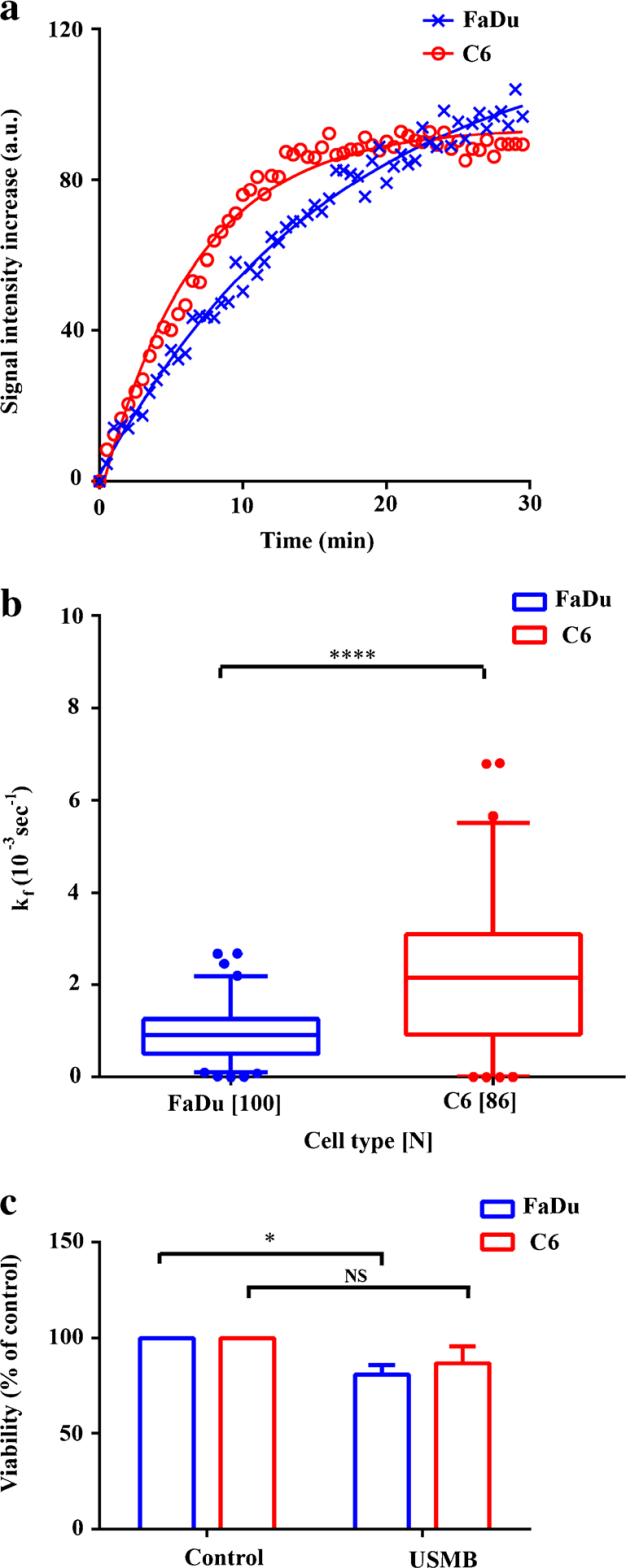
**a** Representative SYTOX Green signal intensity curves over time of FaDu and C6 cells following exposure to 850 kPa ultrasound. **b** Fluorescence rate constants (*k*_f_) versus acoustic pressure for FaDu and C6 cells. Cells were exposed to ultrasound in the presence of 2 μM SYTOX Green. *Whiskers* represent the 5–95 % percentile. **c** Viability of FaDu and C6 cell USMB treatment, measured via MTS assay. *Bars* represent mean + standard deviation (*N* = 3). *NS* not significant. *One asterisk* indicates *p* < 0.05, *three asterisks* indicate *p* < 0.001, and *four asterisks* indicate *p* < 0.0001.

To investigate if USMB exposure of different cell lines resulted in different fluorescence kinetics, C6- and FaDu cells were exposed to USMB at identical experimental conditions. C6 cells reached maximum fluorescence intensity after ∼15 min, while this was >30 min for FaDu cells (Fig. 6a). In fact, the median *k*_f_ of C6 cells was 2.4-fold higher than the *k*_f_ of FaDu cells (Fig. 6b). This long fluorescence signal enhancement was also observed in the swept field confocal microscopy experiments, even though the FCFM used higher acoustic pressures. Moreover, we found no relation between acoustic pressure and fluorescence kinetics in FaDu and C6 cells, while C6 cells showed consistently faster kinetics than FaDu cells at each pressure (Suppl. Fig. 1).

Also in chemically permeabilized cells, C6 cells showed faster fluorescence kinetics than FaDu cells (Suppl. Fig. 2). This suggests that the differences between these cell lines cannot be solely attributed to different susceptibility to USMB exposure, e.g., different pore resealing kinetics.

The long-term viability of FaDu and C6 cells was verified via MTS assay, to rule out that the measured SYTOX Green uptake which was the result of irreversibly sonoporated cells. Fig. 6c demonstrates that USMB exposure of FaDu cells resulted in a viability that was slightly, though significantly, lower than control cells (*p* < 0.05), while the viability of C6 cells was not hampered by USMB (*p* > 0.05). This demonstrates that USMB-induced SYTOX Green uptake measured by FCFM was primarily the result of reversible membrane permeability.

## Discussion

The goal of this study was to characterize the fluorescence signal enhancement after intracellular uptake of SYTOX Green, with regard to the effects of laser duty cycle, dye concentration, and cell line. First, we observed that USMB-induced pore formation resulted in intracellular signal enhancement of SYTOX Green, spreading throughout the cell and ultimately accumulating in the nucleus. By systematically varying experimental parameters, we have demonstrated that the fluorescence kinetics were substantially influenced by laser duty cycle, dye concentration, and cell line.

Membrane impermeant intercalating model drugs like SYTOX Green [12], PI [23], or TOTO-3 [24] are widely used to study USMB-induced intracellular uptake. Due to their large fluorescence enhancement upon binding to nucleic acids, these model drugs display high intracellular and very low extracellular signal intensity, making them useful agents to study cellular internalization.

However, problems emerge when studying the kinetics of intracellular model-drug uptake, as the fluorescence enhancement reflects not only cellular uptake but also intracellular diffusion, nuclear accumulation, and DNA binding. We observed that USMB-induced pore formation resulted in a local entry of SYTOX Green into the cell, similar to previous work with SYTOX Green [25] or PI [10]. Moreover, others have linked the location of a specific microbubble with the location of cellular entry of PI [6, 17, 18]. In our studies, intracellular SYTOX Green signal was seen to spread throughout the cell and ultimately accumulated in the nucleus, which involves intracellular diffusion and DNA binding. The nuclear fluorescence signal increased for more than 9 min, while the cytosolic signal intensity reached a maximum after 15 s. Recently, Helfield *et al.*, made a similar observation, that the cytosol reached maximum fluorescence intensity much faster than the nucleus after USMB exposure in the presence of PI [26]. The fluorescence kinetics of the cytosol may thus hold a more direct association with the membrane permeability, e.g., pore resealing, whereas the nuclear fluorescence kinetics is influenced by additional processes, such as intracellular diffusion and nuclear accumulation. However, the fast plateau of fluorescence intensity in the cytosol may also be influenced by saturation of cytosolic nucleic acids.

Furthermore, signal intensity was much higher in the nucleus compared to the cytosol, most likely as a result of the higher nucleic acid concentration in the nuclear compartment, which can thus bind larger quantities of SYTOX Green. When cellular signal intensity enhancement following USMB treatment is monitored in low spatial resolution, such as with FCFM, the signal from the nucleus is dominant. Therefore, it is not surprising that fluorescence kinetics of the cellular signal on FCFM and the nuclear signal on the swept field confocal microscope were highly similar. The swept field confocal microscope allows us to monitor SYTOX Green signal enhancement with subcellular precision, but there were only a few cells that showed a clear diffusion pattern as shown in Fig. 2. FCFM on the other hand allows studying fluorescence kinetics on a larger scale, typically a population of hundreds of cells at a time.

Fluorescence kinetics were substantially influenced by the laser duty cycle. Although SYTOX Green shows relatively low photobleaching [27]; the continuous laser induced a sixfold enhancement of the photobleaching rate, which resulted in a considerable overestimation of the fluorescence kinetics, that could not be corrected by using a three-compartment model fit of the signal. Therefore, we preferred to minimize the effect of photobleaching by recording the fluorescence signal enhancement with a pulsed laser only. Obviously, the frame rate of pulsed recordings must be high enough to sample the fluorescence signal enhancement. Alternatively, anti-fading agents could be employed to reduce photobleaching [28], but the effect of this approach was not tested in this study.

We found similar fluorescence kinetics between SYTOX Green and PI (Suppl. Fig. 3), while others have reported faster fluorescence kinetics for PI [17, 18]. This is most likely due to the higher PI concentration used in these studies. For example, Fan *et al.* used an extracellular PI concentration of 100 μM and demonstrated that cellular PI signal intensity increased for 1 to 5 min post-sonication before reaching maximum signal intensity. This is in line with the concentration-dependent fluorescence kinetics found in our study, i.e., *k*_f_ = 0.2 (±0.03) × 10^−3^ s^−1^ μM^−1^, where 100 μM dye would result in a fluorescent rate constant of 20 × 10^−3^ s^−1^. This corresponds to reaching 67 % of the maximal fluorescence intensity in 50 s, a similar kinetic pattern as observed by Fan *et al*. Nevertheless, they attributed the time window of fluorescence signal enhancement to the time needed for pore resealing, while assuming instantaneous binding of PI to cellular RNA or DNA [17]. More recently, van Rooij *et al.* also showed fast uptake kinetics of PI following USMB (<2 min), while using a much lower concentration of PI (37 μM) [19]. Similar to Fan *et al.*, they related the time of PI signal enhancement after sonication to pore resealing and attributed signal enhancement beyond 120 s to cell death, which, based on our findings, may also occur in viable cells.

Although the time window of PI signal enhancement following ultrasound exposure has been associated with pore resealing, we demonstrated that this is primarily dependent on experimental parameters, such as laser duty cycle and dye concentration. Fluorescent signal enhancement is much slower at lower dye concentrations, which has been interpreted in some cases as membrane pores being open for much longer. Still, the uptake kinetics of model drugs can be studied using fluorescence intensity enhancement when using identical experimental conditions throughout experiments, but researchers should be aware of the influence of the experimental parameters on the apparent uptake kinetics.

Direct microscopic observations of ultrasound-induced membrane pores have shown that resealing occurs within seconds up to a minute [29]. Therefore, we speculate that during the time that pores are open, SYTOX Green molecules enter the cell, while the pore reseals within a minute. The quantity of intracellular SYTOX Green depends primarily on the extracellular concentration. Subsequently, SYTOX Green diffuses throughout the cell and accumulates in the nucleus by binding to DNA. The rate of diffusion is influenced by the concentration, in accordance with Fick’s law. Similarly, the statistical nature of DNA intercalator binding makes that this rate is influenced by the concentration of the intercalator as well as the number of available binding sites. Furthermore, additional SYTOX Green may enter the cell after the pores have resealed, e.g., by endocytosis [11, 30]. Together, these events result in the signal intensity enhancement, which can last much longer than the time during which the pores remain open.

Alternative methods may also be explored to investigate the uptake kinetics of (model) drugs following USMB therapy. Non-intercalating model drugs like Green Fluorescent Protein (GFP) or FITC-dextrans can be used to exclude the effect of nucleic acid binding. However, as these dyes display high background signal, they are difficult to use for monitoring USMB-induced intracellular influx in real time. In addition, such an approach will face similar limitations as this study, including the semi-quantitative nature of the optically measured kinetic data. Alternatively, intracellular (model) drug concentrations can be quantitatively measured and validated at discrete timepoints, i.e., not in real-time, post-sonication by, e.g., HPLC, but for fluorescent intercalating compounds, the dependence of the HPLC detection on fluorescence is confounding. Furthermore, the transient membrane invaginations resulting from USMB can be studied by transmission electron microscopy or scanning electron microscopy [13], which provide useful insight in the structural changes of the membrane and the timescale of membrane recovery. However, these techniques do not allow monitoring (model drug) transport across the membrane. Lastly, instead of measuring USMB-induced model drug influx, the efflux of an intracellular dye, such as GFP, can be measured by fluorescence microscopy, which can be associated with pore resealing and exocytosis [31].

## Conclusion

This study demonstrated that the kinetics of fluorescence enhancement following ultrasound-induced fluorescent model drug uptake were not only associated with membrane pore resealing but also with intracellular diffusion and accumulation in the nucleus. In addition, we showed that the fluorescence kinetics were substantially influenced by experimental parameters, such as the laser duty cycle, dye concentration and cell line. Therefore, as SYTOX Green fluorescence enhancement is governed by several mechanisms and largely influenced by experimental parameters, its fluorescence kinetics should be carefully interpreted before relating it to biological processes, such as pore resealing. Specifically, the results from experiments and studies using different dye concentrations or fluorescence imaging systems should not be compared directly.

## Electronic Supplementary Material

ESM 1 (PDF 430 kb).
