# Supplementary material for: Dynamic Fluorescence Microscopy of Cellular Uptake of Intercalating Model Drugs by Ultrasound-Activated Microbubbles
Source: Mol Imaging Biol. 2017 Feb 17;19(5):683–93. doi: 10.1007/s11307-016-1042-x (PMC5574961; doi:10.1007/s11307-016-1042-x)
Supplement: Supplementary file 1 — (PDF 430 kb) [file 11307_2016_1042_MOESM1_ESM.pdf]

Electronic Supplementary Material

Dynamic Fluorescence Microscopy of Cellular Uptake of Intercalating Model Drugs by Ultrasound-Activated Microbubbles

Journal: Molecular Imaging and Biology

Lammertink B.H.A.[1], Deckers R.[1], Derieppe M.[1,2], De Cock I.[3,4], Lentacker I.[3], Storm G.[5,6], Moonen C.T.W.[1], Bos C.[1]

[1] Imaging Division, UMC Utrecht, Utrecht, The Netherlands

[2] Present address: Department of Radiology, Leiden University Medical Central, Leiden, The Netherlands

[3] Department of Pharmaceutics, Ghent University, Ghent, Belgium

[4] Present address: Department of Bioengineering, University of Washington, Seattle, USA

[5] Pharmaceutics Department, Utrecht University, Utrecht, The Netherlands

[6] Targeted Therapeutics, MIRA Institute, University of Twente, Enschede, The Netherlands

To whom correspondence should be addressed: C. Bos (c.bos@umcutrecht.nl), University Medical Center Utrecht, Imaging Division, Heidelberglaan 100, 3584 CX Utrecht, The Netherlands. Tel: +31(0)887550279. Fax: +31(0)887555850

Materials & Methods

Data analysis

The photobleaching rate constant k_{pb} was evaluated in chemically permeabilized cells by fitting the fluorescence intensity profiles with a single-exponential decay:

$$I(t) = I_0 e^{-k_{pb}t}$$

Where I_0 is the fluorescence intensity at the beginning of the acquisition, k_{pb} is the photobleaching rate constant and t is the time.

The fluorescence intensity curve following SYTOX Green uptake was fitted with a 2-compartment model, as the photobleaching was low when using the pulsed laser recordings. The fluorescence signal over time was described by the following formula:

$$I(t) = I_\infty\left[1 - e^{-k_f(t-T)}\right]$$

Where I_∞ is the maximal signal enhancement, T the time of fluorescence signal onset, t the time in seconds and k_f the rate constant at which fluorescence approaches equilibrium. Since continuous laser recordings included more photobleaching than the pulsed laser, they were also fitted with a 3-compartment model [28]. Fluorescence rate constants (k_f) were calculated for individual FaDu and C6 cells following ultrasound-induced SYTOX Green uptake. In the spectrofluorometry experiments, k_f values were calculated for a single well, representing a population of cells.

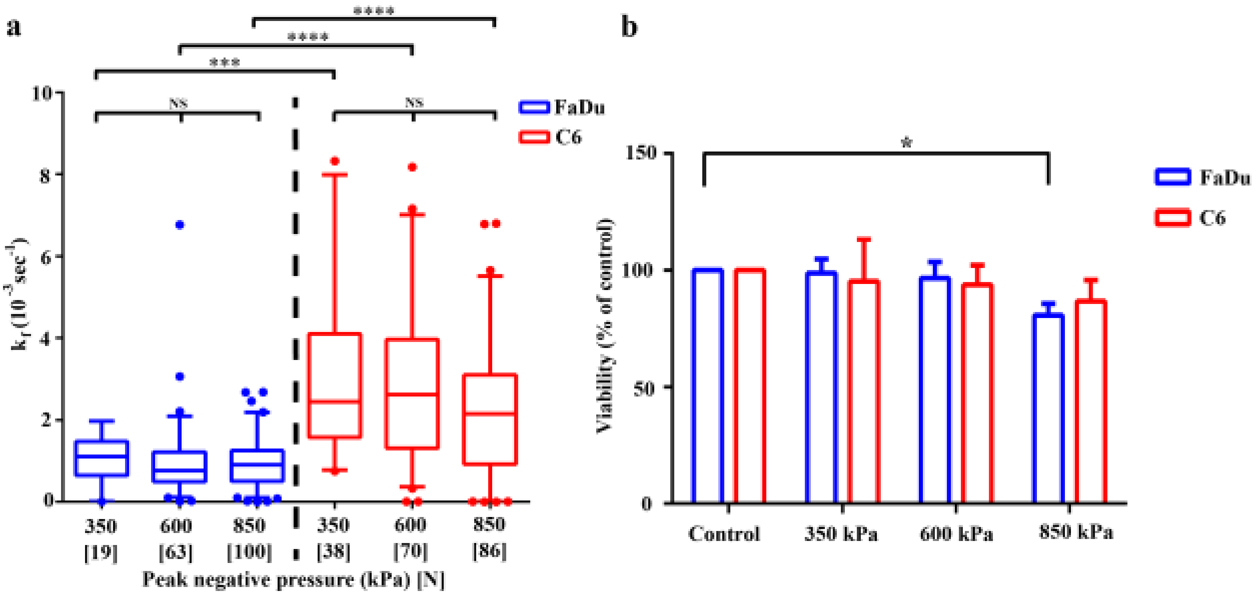

Supplementary Fig. 1: (a) Fluorescence rate constants (k_f) versus acoustic pressure for FaDu and C6 cells. Cells were exposed to ultrasound in the presence of 2 µM SYTOX Green. Whiskers represent the 5 - 95% percentile. (b) Viability of FaDu and C6 cells USMB treatment, measured via MTS assay. Bars represent mean + standard deviation (N=3). NS = not significant, *p < 0.05, *** p < 0.001, **** p < 0.0001.

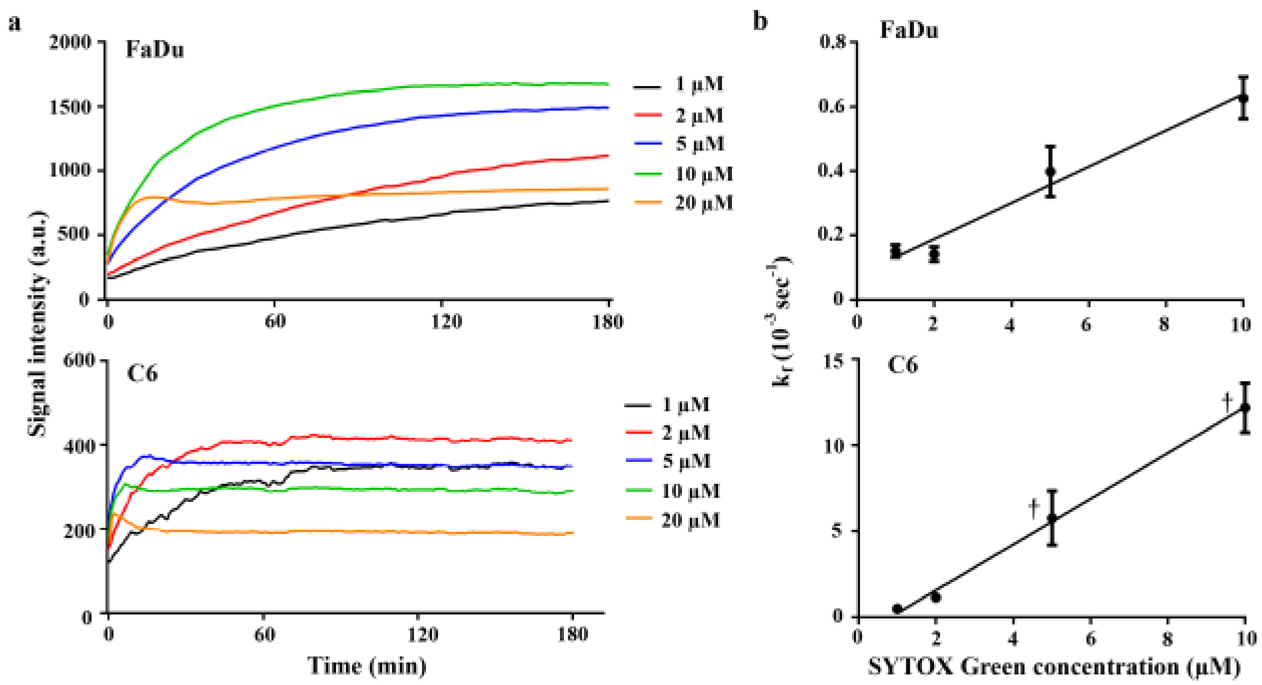

Supplementary Fig. 2: SYTOX Green uptake and fluorescence signal enhancement of chemically permeabilized FaDu cells. (a) Average SYTOX Green signal intensity over time after addition of 1, 2, 5, 10 or 20 µM to permeabilized cells (N=4). (b) Fluorescence rate constants (k_f) of signal intensity after addition of different SYTOX Green concentrations to permeabilized cells as a function of SYTOX Green concentration. Symbols represent mean ± SEM (N=4). † marks the concentrations where quenching was suspected to occur.

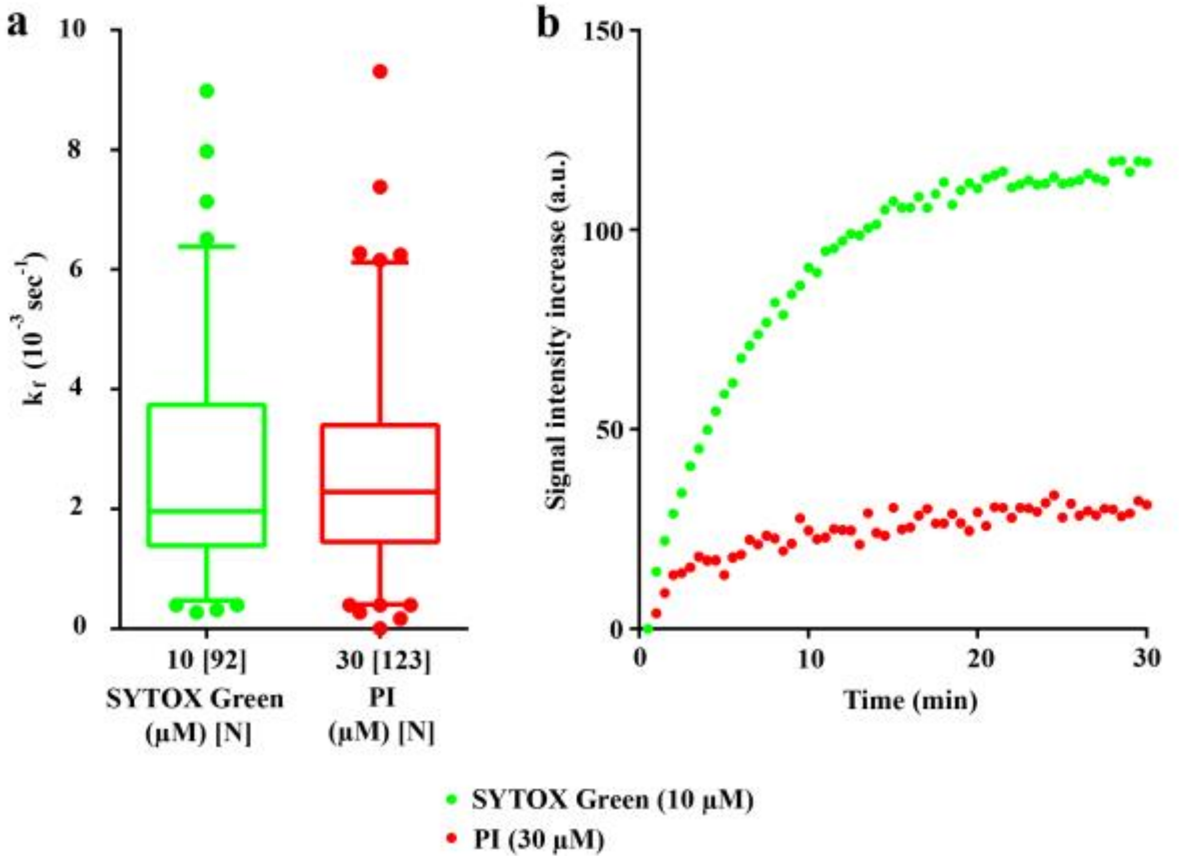

Supplementary Fig. 3: (a) Fluorescence rate constants (k_f) of cells exposed to ultrasound in the presence of SYTOX Green or PI. Whiskers represent the 5 - 95 percentile, N the number of cells per group. (b) Fluorescence signal intensity as a function of time for representative cells that were exposed to ultrasound in the presence of 10 µM SYTOX Green or 30 µM PI.

References

[1] Frenkel V (2008) Ultrasound mediated delivery of drugs and genes to solid tumors. Adv. Drug Deliv. Rev. 60:1193-1208.

[2] Allen TM, Cullis PR (2004) Drug Delivery Systems: Entering the Mainstream. Science 303:1818–1822.

[3] Han HK, Amidon GL (2000) Targeted prodrug design to optimize drug delivery. AAPS PharmSci 2:48-58.

[4] Lentacker I, De Cock I, Deckers R, et al. (2014) Understanding ultrasound induced sonoporation: Definitions and underlying mechanisms. Adv. Drug Deliv. 72:49–64.

[5] Escoffre JM, Piron J, Novell A, Bouakaz A (2011) Doxorubicin Delivery into Tumor Cells with Ultrasound and Microbubbles. Mol Pharm. 8: 799–806.

[6] van Wamel A, Kooiman K, Harteveld M, et al. (2006) Vibrating microbubbles poking individual cells: Drug transfer into cells via sonoporation. J. Controlled Release. 112:149-155.

[7] Tachibana K, Uchida T, Ogawa K, et al. (1999) Induction of cell-membrane porosity by ultrasound. The Lancet 353:1409.

[8] Deng CX, Sieling F, Pan H, Cui J (2004) Ultrasound-induced cell membrane porosity. Ultrasound Med. Biol. 30:519–526.

[9] Meijering BDM, Juffermans LJM, van Wamel A, et al. (2009) Ultrasound and Microbubble-Targeted Delivery of Macromolecules Is Regulated by Induction of Endocytosis and Pore Formation. Circ. Res. 104:679–687.

[10] De Cock I, Zagato E, Braeckmans K, et al. (2015) Ultrasound and microbubble mediated drug delivery: Acoustic pressure as determinant for uptake via membrane pores or endocytosis. J. Controlled Release. 197:20–28.

[11] Zeghimi A, Escoffre JM, Bouakaz A (2015) Role of endocytosis in sonoporation-mediated membrane permeabilization and uptake of small molecules: a electron microscopy study. Phys. Biol.12:66007.

[12] Lammertink BHA, Deckers R, Storm G, et al. (2015) Duration of ultrasound-mediated enhanced plasma membrane permeability. Int. J. Pharm.482:92–98.

[13] Brayman AA, Coppage ML, Vaidya S, Miller MW (1999) Transient poration and cell surface receptor removal from human lymphocytes in vitro by 1 MHz ultrasound. Ultrasound Med. Biol. 25:999–1008.

[14] Lammertink BHA, Bos C, van der Wurff-Jacobs KM, et al. (2016) Increase of intracellular cisplatin levels and radiosensitization by ultrasound in combination with microbubbles. J. Controlled Release. 238:157–165.

[15] Zhong W, Chen X, Jiang P, et al. (2013) Induction of Endoplasmic Reticulum Stress by Sonoporation: Linkage to Mitochondria-Mediated Apoptosis Initiation. Ultrasound Med. Biol. 39:2382–2392.

[16] Derieppe M, Yudina A, Lepetit-Coiffé M, et al. (2013) Real-Time Assessment of Ultrasound-Mediated Drug Delivery Using Fibered Confocal Fluorescence Microscopy. Mol. Imaging Biol. 15:3–11.

[17] Fan Z, Liu H, Mayer M, Deng CX (2012) Spatiotemporally controlled single cell sonoporation. Proc. Natl. Acad. Sci. 109:16486-16491.

[18] Qin P, Xu L, Han T, et al. (2016) Effect of non-acoustic parameters on heterogeneous sonoporation mediated by single-pulse ultrasound and microbubbles. Ultrason. Sonochem. 31:107–115.

[19] van Rooij T, Skachkov I, Beekers I, et al (2016) Viability of endothelial cells after ultrasound-mediated sonoporation: Influence of targeting, oscillation, and displacement of microbubbles. J. Controlled Release. 238:197–211.

[20] Quax PH, van Muijen GN, Weening-Verhoeff EJ, et al (1991) Metastatic behavior of human melanoma cell lines in nude mice correlates with urokinase-type plasminogen activator, its type-1 inhibitor, and urokinase-mediated matrix degradation. J. Cell Biol. 115:191–199.

[21] de Chaumont F, Dallongeville S, Chenouard N, et al. (2012) Icy: an open bioimage informatics platform for extended reproducible research. Nat. Methods. 9:690–696.

[22] Derieppe M, de Senneville BD, Kuijf H, et al. (2014) Tracking of Cell Nuclei for Assessment of In Vitro Uptake Kinetics in Ultrasound-Mediated Drug Delivery Using Fibered Confocal Fluorescence Microscopy. Mol. Imaging Biol. 16:642–651.

[23] Fan Z, Kumon RE, Park J, Deng CX (2010) Intracellular delivery and calcium transients generated in sonoporation facilitated by microbubbles. J. Controlled Release.142:31–39.

[24] Deckers R, Yudina A, Cardoit LC, Moonen CTW (2011) A fluorescent chromophore TOTO-3 as a ‘smart probe’ for the assessment of ultrasound-mediated local drug delivery in vivo. Contrast Media Mol. Imaging. 6:267–274.

[25] Leow RS, Wan JMF, Yu ACH (2015) Membrane blebbing as a recovery manoeuvre in site-specific sonoporation mediated by targeted microbubbles. J. R. Soc. Interface. 12:20150029.

[26] Helfield B, Chen X, Watkins SC, Villanueva FS (2016) Biophysical insight into mechanisms of sonoporation. Proc. Natl. Acad. Sci. U. S. A. 113:9983-9988.

[27] Thakur S, Cattoni DI, Nöllmann M (2015) The fluorescence properties and binding mechanism of SYTOX green, a bright, low photo-damage DNA intercalating agent. Eur. Biophys. J. 44:337–348.

[28] Longin A, Souchier C, Ffrench M, Bryon PA (1993) Comparison of anti-fading agents used in fluorescence microscopy: image analysis and laser confocal microscopy study. J. Histochem. Cytochem. 41:1833–1840.

[29] Hu Y, Wan JMF, Yu ACH (2013) Membrane Perforation and Recovery Dynamics in Microbubble-Mediated Sonoporation. Ultrasound Med. Biol. 39:2393–2405.

[30] Derieppe M, Rojek K, Escoffre JM, et al. (2015) Recruitment of endocytosis in sonopermeabilization-mediated drug delivery: a real-time study. Phys. Biol. 12:46010.

[31] Kaddur K, Lebegue L, Tranquart F, et al. (2010) Transient transmembrane release of green fluorescent proteins with sonoporation. IEEE Trans. Ultrason. Ferroelectr. Freq. Control. 57:1558–1567.
